# An efficient miRNA knockout approach using CRISPR-Cas9 in Xenopus^☆^

**DOI:** 10.1016/j.ydbio.2021.12.015

**Published:** 2022-03

**Authors:** Alice M. Godden, Marco Antonaci, Nicole J. Ward, Michael van der Lee, Anita Abu-Daya, Matthew Guille, Grant N. Wheeler

**Affiliations:** a School of Biological Sciences, University of East Anglia, Norwich Research Park, Norwich, NR4 7TJ, United Kingdom; b King Henry Building, King Henry I St, Portsmouth, PO1 2DY, United Kingdom

**Keywords:** miRNAs, Neural crest, Pigment, Craniofacial, CRISPR-Cas9, Xenopus, INDEL, Insertion deletion (mutation), miRNAs, MicroRNAs, KD, Knockdown, KO, Knockout, NC, Neural crest, NF, Nieuwkoop and Faber, sgRNA, single guide RNA, MO, Morpholino

## Abstract

In recent years CRISPR-Cas9 knockouts (KO) have become increasingly ultilised to study gene function. MicroRNAs (miRNAs) are short non-coding RNAs, 20–22 nucleotides long, which affect gene expression through post-transcriptional repression. We previously identified miRNAs-196a and −219 as implicated in the development of *Xenopus* neural crest (NC). The NC is a multipotent stem-cell population, specified during early neurulation. Following EMT, NC cells migrate to various points in the developing embryo where they give rise to a number of tissues including parts of the peripheral nervous system, pigment cells and craniofacial skeleton. Dysregulation of NC development results in many diseases grouped under the term neurocristopathies. As miRNAs are so small, it is difficult to design CRISPR sgRNAs that reproducibly lead to a KO. We have therefore designed a novel approach using two guide RNAs to effectively ‘drop out’ a miRNA. We have knocked out miR-196a and miR-219 and compared the results to morpholino knockdowns (KD) of the same miRNAs. Validation of efficient CRISPR miRNA KO and phenotype analysis included use of whole-mount *in situ* hybridization of key NC and neural plate border markers such as *Pax3*, *Xhe2*, *Sox10* and *Snail2*, q-RT-PCR and Sanger sequencing. To show specificity we have also rescued the knockout phenotype using miRNA mimics. MiRNA-219 and miR-196a KO’s both show loss of NC, altered neural plate and hatching gland phenotypes. Tadpoles show gross craniofacial and pigment phenotypes.

## Highlights

• We have developed a novel CRISPR-Cas9 approach to KO miRNAs.
• Loss of miR-196a leads to loss of pigment phenotypes.
• Loss of miR-219 leads to craniofacial phenotypes.
• MiRNAs can affect the development of Xenopus neural crest.

## Graphical abstract

**Figure:**
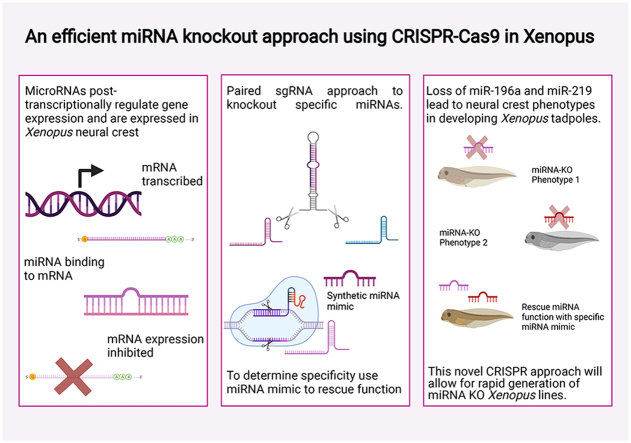

## 1 Introduction

MiRNAs are short non-coding, single stranded RNAs, approximately 20–22 nucleotides in length (Alberti and Cochella, 2017; Lee et al., 1993; Shah et al., 2017). MiRNAs are initially transcribed by RNA polymerase II as a pri-miRNA stem-loop structure from the genome, which undergoes processing to form a mature miRNA (Agarwal et al., 2015; Alberti and Cochella, 2017; Bartel, 2004; Inui et al., 2010).

MiRNAs are highly conserved between species with many orthologues discovered (Bartel, 2004). The miRNA database and repository, miRbase, currently has 2,656 mature miRNA sequences across all species. It is thought that there are >2,300 different miRNAs in humans alone (Alles et al., 2019). Recent reports suggest that 60% of all protein coding genes in mammals are regulated by one or more miRNAs (Li et al., 2018). Within the human genome, it is estimated that up to 2% of genes encode for miRNAs (Miska, 2005). MiRNAs are implicated in development of various tissues in vertebrates, including chick, mouse, frog and fish (Mok et al., 2017; Ward et al., 2018); as well as in invertebrates like the worm and fruit fly (Chandra et al., 2017). Efficient methods to KO one or more miRNAs are therefore required.

The NC has the potential to differentiate into many different cell types and it contributes to many tissues. The NC can migrate all over the body and become parts of the peripheral nervous system, craniofacial skeleton and pigment (Aoto et al., 2015; Cheung and Briscoe, 2003; Hatch et al., 2016). How this occurs depends on niches and environments that have the right cocktail of gene expression patterns, signals or transcription factors (Sauka-Spengler and Bronner-Fraser, 2008). MiRNAs have been suggested to play a role in NC development with Dicer KD experiments in mouse leading to NC cell death by apoptosis (Zehir et al., 2010). We have found that miR-219 and miR-196a are enriched in NC tissue, with miR-219 almost exclusively expressed in NC explants; others have also identified miR-196a implicated in eye development and NC through morpholino miRNA-KD experiments (Gessert et al., 2010; Ward et al., 2018).

CRISPR in recent years has been increasingly used in manipulating gene expression. CRISPR-Cas9 utilizes a highly specific targeted nuclease to induce genomic editing by non-homologous end joining or homology-directed repair. CRISPR is an efficient technology that can rapidly generate KO samples for analysis (Ran et al., 2013). In *X. tropicalis,* Nakayama and colleagues laid the foundation and set out a simple CRISPR pipeline and use of mutations in the tyrosinase gene to generate albinism phenotypes, targeting the start codon, leading to frameshift mutation and KO (Nakayama et al., 2013). CRISPR can be used to analyse gene function, and to replicate human disease mutations to generate mosaic targeted mutant F0’s and lines in *Xenopus* embryos (Feehan et al., 2019; Macken et al., 2021; Naert et al., 2017, 2020; Naert and Vleminckx, 2018; Nakayama et al., 2013).

As part of our ongoing work of looking at miRNAs in NC development we have developed a novel method to KO miRNAs quickly and efficiently in *X. tropicalis* embryos and analyse the phenotype generated transiently in the F0 population. Using this method we have begun to more clearly investigate the role of miR-196a and miR-219 in NC development.

## 2 Results and discussion

### 2.1 miRNA expression profiling

**Fig. 1:**
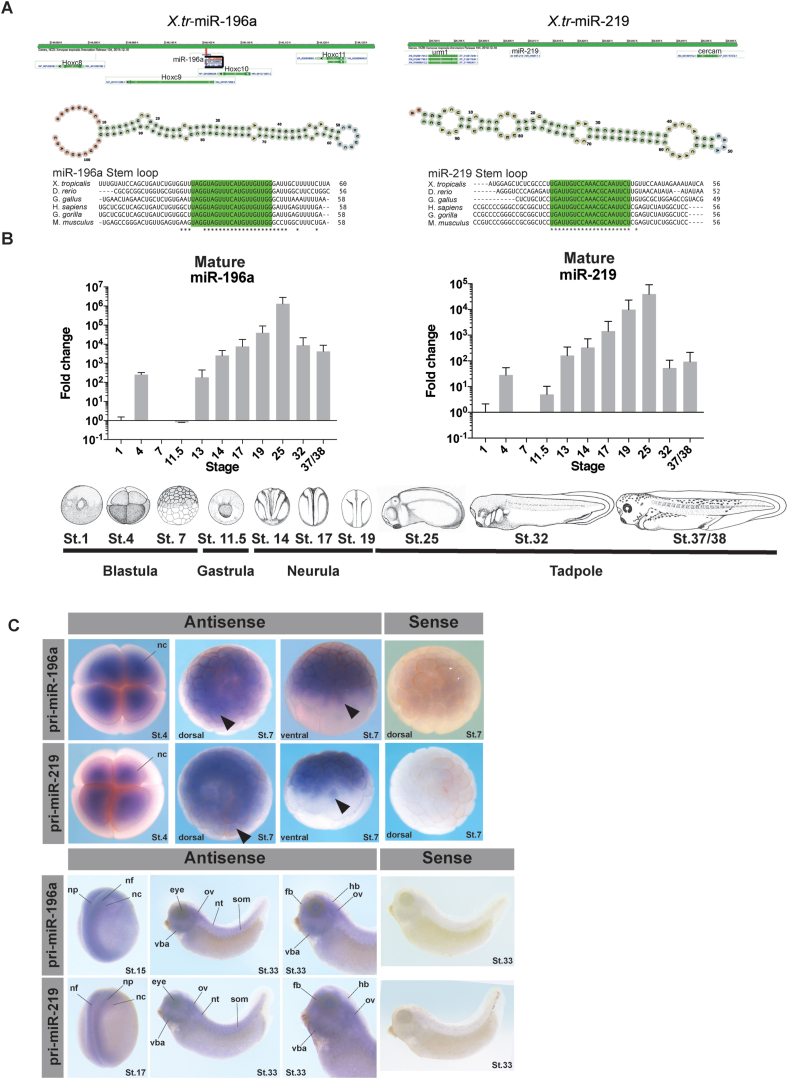
**Conservation, location, spatial and temporal expression of miR-196a and miR-219 in *X. tropicalis***. *(A)* miRNA genomic locations and stem-loop structures of miRNAs. Bottom-conservation of mature miRNAs as indicated by “∗”’s. miRNA stem loop structures were predicted computationally using Vienna RNA fold tool: http://rna.tbi.univie.ac.at/forna/forna.html?id=RNAfold/vCiQTz5Wd4&file=cent_probs.json (B) *X. laevis* developmental profile of miR-196a and miR-219 by qRT PCR. Fold change is represented as mean ​± ​SD normalised to snU6 at St.1, and biological replicates with undetermined values are excluded. Ten embryos (three biological replicates) were pooled to extract total RNA for cDNA synthesis. For each biological replicate three technical replicates were conducted. *Xenopus* embryo pictures at Nieuwkoop and Faber stages are shown. The neural crest generally begins to appear at NF st. 12.5/13. (C) Spatial expression of pri-miR-196a and pri-miR-219 by whole-mount *in situ* hybridisation in X. *tropicalis* embryos with sense and anti-sense expression. Embryos show expression from St.4 with global expression in NC, at St.7 expression is seen in cell nucleus, at neurula stages expression is in NC and neural tissues, and at tadpole stages can be seen in craniofacial tissues. Abbreviations: nf-neural fold, np-neural plate, nc- NC, vba-ventral branchial arches, ov-otic vesicle, nt-neural tube, som-somites, fb-forebrain, hb-hindbrain.

We previously identified miRNAs expressed in NC tissue through RNA-sequencing experiments on Wnt/Noggin induced animal-caps (Ward et al., 2018). Here we focus on two miRNAs identified in our earlier study; miR-196a which is located within the *Hoxc* cluster, in *HoxC9*, and miR-219 which is located intergenically. Both miRNAs have a pri-miRNA stem-loop structure that is highly conserved among the animal kingdom (Fig. 1A). The initial aim was to identify when and where miR-196a and miR-219 are expressed in the developing *Xenopus* embryo. To understand when the miRNAs were expressed, q-RT-PCR was employed. Both miRNAs have a very similar profile with expression peaking initially at Nieuwkoop and Faber (NF) St.4 before dropping at gastrula stages of development and then increasing at late-gastrula and early neurula stages. Expression peaks at St.25 before dropping at tadpole stages (Fig. 1B).

Mature miRNAs are short 20–22 nucleotides long (Bartel, 2004). Due to this they are too short to detect with a standard *in situ* hybridisation probe (Thompson et al., 2007). We have previously used LNA modified *in situ* probes to determine miRNA expression in *Xenopus* embryos; however, LNA probes for miR-196a and miR-219 produced no signal (not shown). Another approach is to generate an antisense pri-miRNA *in situ* probe by PCR from genomic DNA (Walker and Harland, 2008). Using this method we looked at the expression of miR-196 and miR-219. For miR-196a and miR-219, expression is very similar (Fig. 1C). Expression can be seen with the antisense probes but not in sense. At early embryonic stages miRNA expression peaks at St.4 of development. The q-RT-PCR data indicates this at St. 4 and shows no expression at St.7. This is in contrast to the whole mount *in situ* hybridisation data which shows clear expression of miRNA. This could be explained by the fact that the *in situ* hybridisation experiment shows precursory miRNA expression, and q-RT-PCR shows mature miRNA expression. This may explain why the St.7 expression profile for both pri-miR-196a and pri-miR-219 are localized to the cell nucleus (Bartel, 2004). St. 4 expression however is ubiquitous across the upper level of cells, in the dorsal and ventral animal cells (Fig. 1B–C).

At neurula stages, NF St. 15 and 17, expression is seen in neural folds, neural plate and NC. At tadpole stage, NF St. 33, expression can be seen in craniofacial tissues, including NC derivatives, the otic vesicle and ventral branchial arches. Using LNA probes we also observed miR-196a and miR-219 expression in chick embryos in the neural tube, neural tissue and some expression in NC (Suppl. Fig. 1).

### 2.2 Developing and employing CRISPR-Cas9

CRISPR-Cas9 approaches have been developed in many species including *Xenopus* (Nakayama et al., 2013). To KO a miRNA in *X. tropicalis*, the technical limitation of generating a viable embryo with a clean KO is that designing a sgRNA close to or in the miRNA is difficult due to their small size. In addition an insertion/deletion (INDEL) mutation could lead to generation of a novel miRNA as well as losing the orginal miRNA (Bhattacharya and Cui, 2017). This was the main concern when an individual sgRNA was implemented to mutate the mature miRNA (Suppl. Fig. 4). Here it was shown that one sgRNA could not significantly disrupt the mature miRNA enough to alter the processing or structure of the pri-miRNA, and thus the mature miRNA would like have been potentially expressed or have become a novel miRNA with an altered sequence to its parent.

**Fig. 2:**
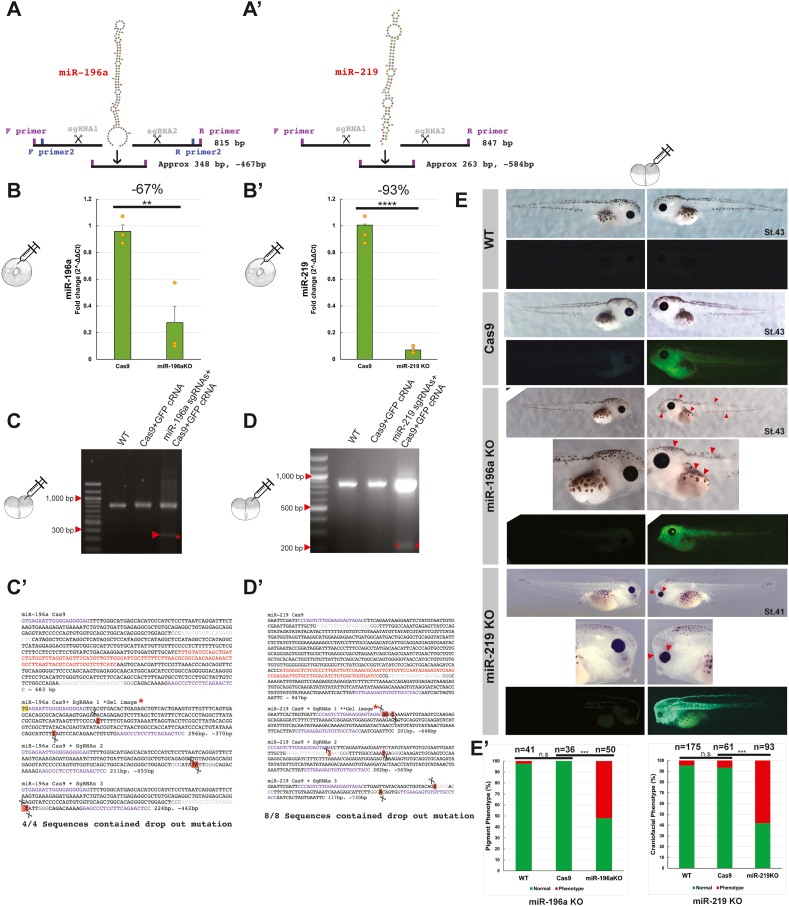
**CRISPR-Cas9 approach for knocking out miRNAs in *X. tropicalis* and validation strategies.** (A-A’) Schematic showing the approach taken with use of two sgRNAs for miR-196a (A) and miR-219 (A’). (B) Q-RT-PCR validation of miR-KO, with individual data points from biological repeats shown in orange. MiR-196a KO showed a 67% reduction in expression (B), and miR-219 KO showed a 93% reduction in expression following CRISPR-Cas9 treatment (B’). Embryos were injected at the 1 ​cell stage, bar charts show mean_+/− S.E.M. Experiments were conducted with biological and technical triplicate. (C–D) PCR/nested PCR validation of gDNA miRNA regions from embryos injected into one cell at 2 ​cell stage of development, with KOs showing an extra smaller band in the fourth lane of each gel. (C’-D’) Sanger sequencing validation of miRNA KOs and CRISPR events. Cas9+GFP control samples were also harvested, genomic DNA extracted, PCR amplified and subcloned. Purple text highlights primers used for cloning, red text shows miRNA stem-loop. Yellow highlight shows a mis-match, and red highlight with scissor icons show where CRISPR events occurred, grey text shows sgRNA. WT and Cas9 sequences show miRNA WT sequence, whereas Cas9+sgRNAs show 3 repeats of mutated sequences, with significantly shorter sequences. (E) Phenotype analysis of miRNA KO embryos, representative embryos are shown. Embryos were co-injected with CRISPR-reagents a GFP capped RNA tracer into one cell at a two cell stage of development. Embryos are imaged on left and right sides. WT had no injection, Cas9 protein was co-injected with GFP cRNA and miR-KO were pairs of sgRNAs, Cas9 protein and GFP cRNA tracer. The fluorescent side of miR-196a KO embryo red arrows indicate a pigment phenotype, and for miR-219 KO, the red arrows indicate a strong craniofacial phenotype, with smaller eyes, branchial arch and flattened face features. (E’) Bar charts show count data of yes/no phenotypes for miR-196a KO (pigment loss) and miR-219 KO (craniofacial disfigurement), with chi-squared tests for statistical significance. There was a significant difference between and Cas9 and miR-196a KO groups p ​= ​2.22 ​× ​10^-7^ and between Cas9 and miR-219 KO p ​= ​1.1 ​× ​10^-10^. Embryo phenotypes were blind counted on three biological repeats on embryos from different *Xenopus* parents.

Other ways to KD miRNA expression by CRISPR include targeting the DROSHA and DICER processing sites within the miRNA under study (Chang et al., 2016). Again, this is not always possible due to sgRNA design limitations due to the NGG PAM design for Cas9 used in this study (Wilson et al., 2018). By designing sgRNAs flanking the stem-loop of the miRNA, it was predicted they would simultaneously create double-stranded breaks in the genome to “drop-out”, the entire miRNA stem-loop, and give a clean miRNA KO (Fig. 2A and Table 1).

**Table 1:** SgRNA sequences used. Common oligo taken from (Nakayama et al., 2013).

SgRNA (sgRNA) Oligo	Sequence 5’ to 3’
sg219-5	taatacgactcactataGGTGAATTTTCCACAGCAATgttttagagctagaa
sg219-9	taatacgactcactataGGGTCTTCAGAATCAGCGACgttttagagctagaa
sg196-4	taatacgactcactataGGGAGGCTTCTCAGAATATTgttttagagctagaa
sg196-7	taatacgactcactataGGGAGCCTATGGAGCCATATgttttagagctagaa
Common oligo (reverse primer)	AAAAGCACCGACTCGGTGCCACTTTTTCAAGTTGATAACGGACTAGCCTTATTTTAACTTGCTATTTCTAGCTCTAAAAC

To be confident the miRNAs were knocked out, pairs of sgRNAs, Cas9 protein and GFP cRNA were co-injected into *X. tropicalis* embryos into both blastomeres at the 2-cell stage to target whole embryo (Fig. 2A). Embryos expressing GFP on both sides were selected, and 5 ​St.14 neurulas were pooled to produce independent biological repeats. RNA was harvested to analyse miRNA expression by q-RT-PCR to evaluate sgRNA efficiency. The results showed that expression of both miRNAs was reduced in the treated samples, compared to control embryos injected with Cas9 protein and GFP cRNA tracer. MiR-196a sgRNAs resulted in a 67% reduction of miR-196a expression and miR-219 sgRNAs reduced miR-219 expression by 93% as compared to control samples (Fig. 2B). Differences in efficiency could be due to the nature of CRISPR (Ran et al., 2013).

Next, we identified the types of INDEL generated using the two guide-RNAs approach. Genomic DNA was extracted from individual *X. tropicalis* embryos injected with CRISPR reagents and a GFP capped RNA tracer into one blastomere at 2-cell stage of development. Cas9 ​+ ​tracer was used as a negative control. PCR was carried out to amplify the stem-loop of the miRNA (Fig. 2A). As expected, we detected wild-type (WT) miRNA in all samples as only half the gDNA could have been mutated. For the miR-KO samples an extra smaller band was seen on the gel (Fig. 2C and D). For miR-196a, the WT miRNA band is 815 bp. The sgRNAs should lead to the deletion of 467 bp and release a fragment of approximately 300 bp if a CRISPR event was successful. For miR-219 the WT miRNA should be 847 bp, and the CRISPR-released fragment is expected to be 260 bp or smaller, as seen in the gel (Fig. 2 C & D). Amplified products were then gel extracted and sent for Sanger sequencing (Fig. 2 C’ & D’). For miR-196a a nested PCR was carried out using primer set 3 (Table 2). Stem-loops for each miRNA are shown by red text (Fig. 2 C’ & D’) and as expected the “drop-out” bands do not contain the miRNA. This confirms the successful CRISPR deletion of miRNA stem-loops. The WT and Cas9 control group bands were also extracted and sent for sequencing. The sequencing all showed wild-type miRNA sequences for these. This to our knowledge, is the first time this approach with two sgRNAs has been used in *Xenopus* embryos, though Kretov et al. (2020) do report a similar approach in Zebrafish (Kretov et al., 2020) .

**Table 2:** Table of primers and sequences. Primers used for sequencing and PCR of genomic DNA. Designed using Primer3.

Primer	Sequence 5’ to 3’
219 R3	GGTAGGCAACACACTCTTCAAC
219 R4	GAAGGCTGTATTTTAGCCCTGGC
219 6F	CCCAGTCTTGGAAGGAGTAGAC
196 F3	GTGAGAATTGGGGAGGGGAG
196 R3	AGGAGTTCTGAAGGAGGGCTTC
196 F7	CAGCCCAGCACTTACAGGTT
196 R7	GGAGTTCTGAAGGAGGGCTT
196 F5	TTCAGGACACCTTGTCTGGC
196 R5	TGAGCTTTCCGGTTTAGGGG

Some embryos were left to develop into tadpoles for phenotype analysis. Embryos were targeted with CRISPR reagents on one side only for comparison with the non-injected side as an internal control. WT and Cas9 embryos look morphologically normal on both sides. However, miR-196a tadpoles on the “crispant” (CRISPR-mutated) side show pigment phenotypes, with a reduction in pigment seen along the cranial, dorsal and medial abdominal regions, as indicated by red arrows (Fig. 2E). MiR-219 tadpoles show gross craniofacial impairments; with smaller eyes and flattened anterior nasal region, as shown by the red arrows (Fig. 2E). Blind counts of the phenotypes showed that over 50% of embryos carried the respective pigment and craniofacial phenotypes (Fig. 2E’). These phenotypes suggest a possible role for miR-196a and miR-219 in NC development (Collazo et al., 1993; Lukoseviciute et al., 2018; Petratou et al., 2021; Scerbo and Monsoro-Burq, 2020; Spokony et al., 2002).

To validate the specificity of the miRNA CRISPR KO, a novel rescue experiment was developed (Suppl. Fig. 5). To do this a miRNA mimic was used to rescue the miRNA KO. As a control experiment for this, a control miRNA mimic was used. This was C. *elegans* miRNA, cel-miR-39-3p, as recommended by the manufacturer. This was chosen as a miRNA that should not have an effect on Xenopus development, and not rescue our miR-219 KO.

In Supp Fig. 5A, miRNA mimic was tested for phenotype on its own at a dose of 11 ​μM to see if overexpression of miRNA would induce phenotype. For miR-219 mimic this led to minor craniofacial phenotypes as indicated by the black arrow (Suppl. Fig. 5A and B). In contrast, the overexpression of control mimic miRNA alone did not have a significant impact on embryo phenotype (Suppl. Fig. 5B). To rescue the CRISPR KO of miR-219 the miR-219 miRNA mimic was co-injected into the embryo along with the CRISPR reagents (Suppl. Fig. 5C and D). Craniofacial phenotype were observed in miR-219 KO and miR-219 KO ​+ ​control mimic groups. The phenotype was not observed as much in miR-219 KO ​+ ​miR-219 mimic group and is indicative of a successful rescue of loss of miR-219. This suggests specificity for our novel miRNA KO and rescue experimental design. This is significant as the only currently known reported use of a miRNA mimic used to rescue phenotypes in developing embryos was reported in Zebrafish to overexpress miR-9 to alter and reduce mRNA expression of VEGF-alpha (Madelaine et al., 2017).

MiRNAs can be produced from independent genes or encoded in intronic regions of the genome. They are most commonly found in intergenic, intronic regions of the genome, and rarely found in exonic regions (Olena and Patton, 2010). The proposed CRISPR method in this paper works for intergenic and intronic miRNAs, this was not tested on exonic miRNAs, as these are extremely rare, but could be used with caution. We show this by knocking out miR-196a which is located in a *Hoxc* intron and miR-219, which is intergenic (Fig. 1A).

### 2.3 Exploring miRNA phenotypes

**Fig. 3:**
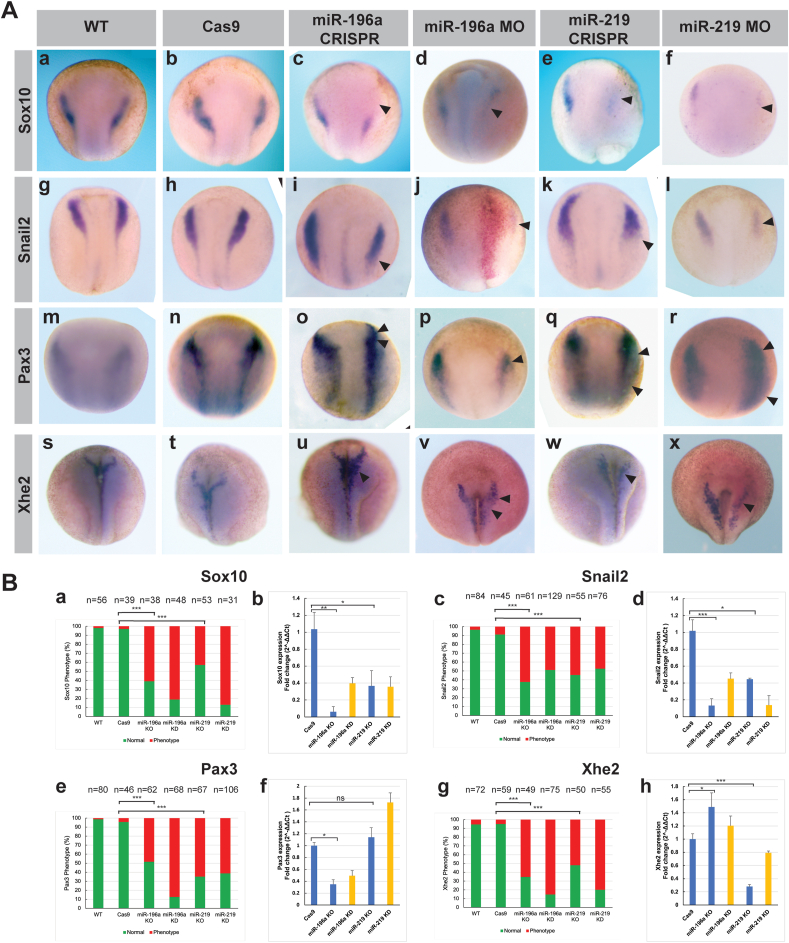
**Analysis of key NC, neural plate and hatching gland markers after miRNA KO and KD.** (A) Whole mount *in situ* hybridisation profiles on neurula stage *Xenopus* embryos of Sox10, Snail2, Pax3 and Xhe2. CRISPR-Cas9 was carried out in *X. tropicalis* embryos with GFP cRNA as a tracer and morpholino-KD was carried out in *X. laevis* embryos with lacZ cRNA as a tracer. Embryos for whole mount *in situ* hybridisation were injected with tracer at 4-cell stage of development into the right dorsal blastomere. Panel A, a-f show Sox10 expression following CRISPR and MO experiments. Panel A g-l show Snail2 expression following CRISPR and MO experiments. Panel A m-r show Pax3 expression following CRISPR and MO experiments. Panel A, s-x shows Xhe2 expression following CRISPR and MO experiments. Overall phenotypes show a reduction of NC and altered neural plate and hatching gland profiles. (B) Phenotype analysis for individual markers. a, c, e and g show count data of yes/no phenotype prescence. Chi-squared statistical tests were carried out on three biological repeats of whole mount *in situ* hybridisation on embryos from different frogs. b, d f and h, Q-RT-PCR results of mRNA expression analysis following CRISPR KO and MO KD. Normalised to U6 expression, RNA was pooled from 5 individual neurula embryos for one biological sample, Q-RT-PCR was carried out with biological and technical triplicates. The Q-RT-PCR data supports phenotypes shown in (A). Panel B, a-b show Sox10 expression following CRISPR KO and MO KD. Panel B, c-d show Snail2 expression following CRISPR KO and MO KD. Panel B, e-f show Pax3 expression following CRISPR KO and MO KD. Panel B, g-h show Xhe2 expression following CRISPR KO and MO KD experiments. Panel B, a,c,e and g show phenotype count data. Panel B, b,d,f and h show q-RT-PCR expression of mRNAs following miRNA KO and KD. Abbreviations for phenotype and q-RT-PCR bar charts in (B): miR-KO refers to CRISPR miRNA KO and miR-KD refers to MO KD of miRNA. Phenotypes for miRNA KD/KO: Sox10 phenotype is a reduction in expression, Snail2 is a reduction/shift in profile, Pax3 phenotype is a shift/reduced profile for miR-196a and an expansion for miR-219 experiments, finally, Xhe2 is an increased profile for miR-196a and a reduced profile for miR-219 experiments respectively. Statistical significance: Sox10 Cas9 vs miR-196a KO p ​= ​4.02 ​× ​10^-8^, Cas9 vs miR-219 KO p ​= ​1.04 ​× ​10^-5^, Snail2 Cas9 vs miR-196a KO p ​= ​6.15 ​× ​10^-9^, Cas9 vs miR-219 KO p ​= ​4.07 ​× ​10^-7^, Pax3 Cas9 vs miR-196a KO p ​= ​7.19 ​× ​10^-7^, Cas9 vs miR-219 KO p ​= ​2.29 ​× ​10^-8^, Xhe2 Cas9 vs miR-196a KO p ​= ​7.19 ​× ​10^-7^, Cas9 vs miR-219 KO p ​= ​2.29 ​× ​10^-8^.

To verify if our miRNAs were implicated in the development of NC, we determined the expression of key markers Sox10, Snail2 for NC, Pax3 for neural plate and Xhe2 for hatching gland (Fig. 3). In addition, we compared the efficiency of our CRISPR KO approach versus morpholino (Table 3) mediated KD of the miRNAs. Control and optimization experiments for miRNA-morpholino KD can be seen in Suppl. Fig. 2.

**Table 3:** Injected morpholino sequence data.

Morpholino	Sequence
miR-196a MO	5’- CAATCCCAACAACATGAAACTACCT-3’
miR-196a Mismatch	5’-CATTGCCAAGAACATCAAAGTACCT-3’
miR-219 MO	5’-AGAATTGCGTTTGGACAATCAAGGG-3’
miR-219 Mismatch	5’ ACAATTGCCTTTCGAGAATCAACGG-3’

For miR-196a, KO and KD led to distinct reduction in Sox10 expression. For Snail2, miR-196 KO and KD led to a reduction and shift in expression. For Pax3 expression a slight reduction and shift in profile was observed and finally Xhe2 expression was expanded following miR-196a KO and KD, (Fig. 3A–B). For miR-219 KO and KD, Sox10 expression was markedly decreased. Like with miR-196 KD, Snail2 expression following miR-219 KD was reduced, but miR-219 KO showed more of a shift in profile with slight reduction. Following miR-219 KO and KD, Pax3 expression was greatly increased and expanded (Fig. 3A–B). Section data showed miR-219 KD led to Pax3 expansion over the superficial ectoderm but not following miR-196a KD (Suppl. Fig. 3). Phenotypes shown by *in situ* hybridisation were more prevalent for miRNA-KD than KO. This could be due to potential mosaicism of CRISPR events seen in F0 embryos thus leading to variable miRNA levels between embryos (Naert et al., 2020).

Q-RT-PCR was conducted to quantify the phenotypic change in expression of the above markers across the whole embryo, and to compare the efficacy of CRISPR experiments in comparison to morpholino (MO)experiments knocking down mRNA expression for neural crest, neural plate border and hatching gland markers (Fig. 3B). Q-RT-PCR was carried out on crispant samples, as described previously, injecting CRISPR reagents into one blastomere at the 1 ​cell stage. For mRNA expression, all the Q-RT-PCR data was in agreement with the *in situ* data including for the miR-219 KO on Pax3, which was shown to be increased in expression though this was not significant (Fig. 3B). Reasons for this could be that the whole embryo was used for RNA extraction. In Suppl.Fig.3 the sections of the Pax3 embryos following miR-219 KD show that the expansion of Pax3 is shifted and limited to the superficial ectoderm (Suppl. Fig. 3). The MO KD q-RT-PCR data seen alongside the CRISPR KO data for miRNAs in Fig. 3B panel b, d, f and h show the same trends in mRNA expression for Sox10, Snail2, Pax3 and Xhe2 respectively. The data indicate that miR-196a and miR-219 could be involved in early regulation of NC development. Additionally the lab has shown Eya1 to be a direct target of miR-219 by luciferase assay (Ward and Wheeler, unpublished results). Using MO KD, q-RT-PCR also showed changes in expression following loss of miRNA (Suppl. Fig. 6). Loss of miR-196a led to loss of Eya1, loss of miR-219 led to enrichment of Eya1; further supporting the work to show Eya1 is a target of miR-219.

The incidence rate of phenotypes can be seen in Fig. 3B a,c,e and g; with the observed phenotypes clearly occurring in the experimental groups. Broadly miRNA KD and KO phenotype incidence were similar between morpholino and CRISPR, although miR-196a morpholino had a higher rate of phenotype incidence for Sox10 and Pax3. Q-RT-PCR profiles match the *in situ* data, with NC markers showing significant decreases in expression, more notably for miR-196a. Pax3 expansion for miR-219 KO was not statistically significant, but miR-196a KO led to significant reduction in expression. Xhe2 showed significant expansion for miR-196a KO and significant decrease in miR-219 KO. These results show that miRNAs are likely to be implicated in the development of the *Xenopus* NC and can be analysed through use of CRISPR to KO miRNAs.

The loss of Sox10 expression shown in Fig. 3A for miR-196a and miR-219 KD and KO supports the phenotypes shown in the tadpoles in Fig. 2E. The tadpole phenotypes for miR-196a show loss of pigment, and for miR-219 show craniofacial abnormalities, which is significant as Sox10 is involved in trunk NC to produce pigmentation (Aoki et al., 2003) and is disrupted in neurocristopathies affecting cranial NC (Devotta et al., 2016). The craniofacial phenotypes in Fig. 2E included flattened fronto-nasal regions, and smaller eyes, as indicated by the red arrows.

Snail2 expression is downregulated following loss of miRNA (Fig. 3A). This could mean that miRNA KO and KD is leading to a loss of NC differentiation, and may help explain the craniofacial phenotypes seen in miR-219 KO tadpoles (Fig. 2E), (Li et al., 2019). The loss of pigment phenotype seen following miR-196a KO (Fig. 2E) is typical of problems in trunk NC development (Abu-Elmagd et al., 2006). This is also supported by Snail2^−/−^ mice that show patchy pigmentation phenotypes (Shi et al., 2011).

The loss of Pax3 seen in Fig. 3A following loss of miR-196a also supports the pigment phenotype seen in Fig. 2E, as Pax3 is essential for the development of pigment (Kubic et al., 2008). Waardenburg syndrome type 1 and 3 are caused by Pax3 gene mutations, and type 2 and 4 are affected by Sox10 and MITF levels and mutations. Pax3 and Sox10 regulate expression of MITF and thus melanocyte development (Bondurand et al., 2000). As expected, our results follow this by showing reduced Pax3 and Sox10 expression following miR-196a loss (Fig. 3A) which led to pigment loss in tadpoles (Fig. 2E). The expansion of Pax3 following loss of miR-219 (Fig. 3A), could be affecting NC specification leading to the loss of NC induction expression of Snail2 and Sox10 seen in Fig. 3A (McKeown et al., 2005; Monsoro-Burq et al., 2005).

The expansion of Pax3 observed in superficial ectoderm (Suppl Fig. 3) following miR-219 KD could also be indicative of increased neural pluripotency (Chalmers et al., 2002). *Xenopus* neural plate border region can give rise to placodal ectoderm, hatching gland and NC. The hatching gland marker Xhe2 demarcates this. Xhe2 expression is affected by Pax3 expression (Hong and Saint-Jeannet, 2007, 2014). MiR-196a and miR-219 KO and KD experiments show altered Pax3 expression states, therefore as expected Xhe2 expression was also affected. In contrast to results seen in (Hong and Saint-Jeannet, 2014), Pax3 expansion does not lead to expanded Xhe2 in our work. This could be due to the other signals that mediate Xhe2 like Zic1. Further work is required to investigate more markers across neural plate and neural deriviatives.

## 3 Conclusions

One drawback of CRISPR gene-editing is finding sgRNAs that are effective especially when studying miRNAs. As mentioned, a challenge with CRISPR experiments is that with a short target sequence the number of sgRNAs that can be designed is limited (Najah et al., 2019). With the advent of more Cas9 nucleases with broader PAM recognition sequences more designs could be generated (Kim et al., 2020). Furthermore new sgRNA design tools are making sgRNA design easier and more robust (Hsu et al., 2021). However, using sgRNAs flanking the miRNA stem-loop expands the potential for identifying and generating optimal sgRNAs. Using a pair of sgRNAs leads to a complete loss of the miRNA in the majority of embryos. The method described is a quick and efficient way to KO specific miRNAs in independent genes or within introns. With the generation of lines of frogs, time would be saved from laborious injections of morpholinos and controls thus more ambitious and technically demanding experiments would be more realistic.

This work shows that miRNAs miR-196a and miR-219 are expressed in NC and neural tissue. Phenotype analysis shows that the miRNAs are important for NC and hatching gland development. This body of work establishes a protocol and controls for CRISPR experiments for knocking out and rescuing miRNAs in embryo development and puts forward CRISPR as not only a tool to rival use of morpholinos in embryo research but also to potentially replace in certain instances.

## 4 Materials and methods

### 4.1 Xenopus husbandry

All experiments were carried out in accordance with relevant laws and institutional guidelines at the University of East Anglia, with full ethical review and approval, compliant to UK Home Office regulations. Embryos were generated as described in (Harrison et al., 2004; Williams et al., 2017). *X. tropicalis* embryos obtained by priming females up to 72 ​h before use with 10 ui chorulon and induced on the day of use with 200ui. Eggs were collected manually and fertilised in vitro. Embryos were de-jellied in 2% L-cysteine, incubated at 23°C and microinjected in 3% Ficoll into 1 ​cell at the 2–4 ​cell stage in the animal pole. Embryos were left to develop at 23°C. Embryo staging is according to Nieuwkoop and Faber (NF) normal table of Xenopus development (Nieuwkoop and Faber, 1967). GFP/LacZ capped RNA for injections was prepared using the SP6 mMESSAGE mMACHINE kit, 5 ​ng was injected per embryo.

### 4.2 CRISPR-Cas9

SgRNAs were designed using CRISPRScan (https://www.crisprscan.org/), (Moreno-Mateos et al., 2015). miRNA sequences were attained from miRbase (http://www.mirbase.org/); under accession numbers: Xtr-miR-219 MI0004873, Xtr-miR-196a MI0004942. SgRNAs were designed up and downstream of the miRNA stem-loop. miRNA stem loop structures were predicted computationally using Vienna RNA fold tool (http://rna.tbi.univie.ac.at/forna/forna.html?id=RNAfold/vCiQTz5Wd4&file=cent_probs.json).

### 4.3 Embryo injection

Embryos were injected using a 10 ​nL calibrated needle. For *X. laevis* 10 ​nL injections, for *X. tropicalis* 4.2 ​nL injections were used. Cas9 protein was added to a 3 ​μL reaction volume, to give a final concentration of 2.4 ​mM (New England Biolabs, #M0646M, EnGen Cas9 NLS 20 ​μM). 300 ​pg of sgRNAs along with 5 ​ng of GFP capped RNA were co-injected into the *X. tropicalis* embryos simultaneously at 2–4 ​cell stage of development. For q-RT-PCR both sides of embryo were targeted, for gene expression and morphological analysis phenotypes 1 side of the embryo was targeted, with embryos injected at 4 ​cell stage into one dorsal blastomere for whole-mount *in situ* experiments and morphological analysis.

### 4.4 CRISPR validation

Embryos were left to develop until tadpole stages and underwent phenotype scoring. Embryos were then frozen on dry ice before genomic DNA extraction. Genomic DNA was isolated using PureLink Genomic DNA Mini Kit, K1820-00 (Invitrogen, California, USA), according to manufacturers guidelines and then quantified using a Nanodrop 1000. Genotyping PCRs were conducted and products underwent gel extraction before subcloning and sanger sequencing. CRISPR rescue experiments utilised an LNA miRNA mimic from Qiagen. They were ordered at 5 ​nmol with no labelling and desalting. Before use they were diluted in 75 ​μL of nuclease free water to give a concentration of 66.7 ​μM and stored in small aliquots at −20^o^C. Qiagen could not provide a molecular weight for the mimic. The approximate molecular weight using the following formula:

Molecular weight ​= ​320.5 X Number of nucleotides of RNA.

For the miR-219 mimic this equated to 6730.5 therefore:

66.7 ​μM ​= ​448.9 ​ng / μL

6.67 ​μM ​= ​44.9 ​ng / μL

1 ​μM ​= ​6.73 ​ng / μL

MiRCURY LNA miRNA mimics were used to replace miRNA in CRISPR miRNA-219 KO embryos in rescue experiments. MiR-219 mimic was used from (Qiagen, 339173 YM0047076-ADA, MIMAT0000276); hsa-miR-219a-5p miRCURY LNA miRNA Mimic, compatible and fully aligning with *Xenopus* miR-219, Xtr-miR-219 sequence: 5'UGAUUGUCCAAACGCAAUUCU. A negative control miRNA mimic recommended by Qiagen was used (Qiagen, 331973 YM00479902-ADA); Negative control (cel-miR-39-3p), sequence 5’UCACCGGGUGUAAAUCAGCUUG. Mimics were used at a final concentration of 11 ​μM dose with CRISPR reagents as described above, or 11 ​μM alone +5 ​ng GFP cRNA tracer.

#### 4.4.1 Morpholinos

Morpholino dose was optimized to 60 ​ng for miRNAs; morpholino and lacZ capped RNA tracer were injected at 4 ​cell stage of embryo development into the right dorsal blastomere.

### 4.5 Phenotype statistical analysis

Chi-squared test for association was used to test phenotype yes or no categories for morpholino injected embryos to see if there was a relationship between two categorical values. Excel was used to collate and tabulate data. IBM SPSS v25 to carry out chi-squared test. Statistical significance is reported as; p ​< ​0.05 ​= ​∗, p ​< ​0.01 ​= ​∗∗, p ​< ​0.001 ​= ​∗∗∗.

### 4.6 CDNA synthesis

MiRCURY LNA RT kit (Qiagen, Cat No./ID: 339340) was used to produce cDNA for q-RT-PCR. 50 ​ng of RNA was used to generate cDNA according to manufacturers instructions. cDNA was produced on a thermocycler with the following programme: 42 ^o^C for 60 ​min and 95 ^o^C for 5 ​min cDNA was diluted 1:40 for q-RT-PCR. CDNA can be stored at −20^o^C. To produce cDNA for mRNA q-RT-PCR the following recipe was used: 500 ​ng of total RNA was added in 9 uL of nuclease free water, plus 2 uL of random primers (Promega, C1181). This was then incubated at 70 ^o^C for 10 ​min. A mastermix was prepared as follows per sample: 4 uL of 5X buffer, 2 uL of DTT, 1 uL of dNTPs, 1 uL of Superscript II (Invitrogen, 18064014), 1 uL of nuclease free water or RNasin (Promega, N2611). qRT-PCR reactions were set up in 10 ​μL volume containing 4 ​μL cDNA, 1 ​μL primer (10 ​μM for standard oligo primers), and 5 ​μL SybrGreen (Applied Biosystems 4309155).

### 4.7 Q-RT-PCR

Embryos were frozen on dry ice before RNA extraction. For miRNA and mRNA quantification total RNA was extracted from five St.14 *X. tropicalis* embryos, embryos were homogenised with a micropestle and RNA was extracted according to manufacturers guidance, Quick-RNA Mini prep plus kit (Zymo, Cat no. R1058). Samples were eluted in 25 ​μL of nuclease free water; RNA concentration and purity quantified on a Nanodrop 1000 and 1 ​μL was checked on a 2% agarose gel. All q-RT-PCR’s were performed with triplicate biological and technical repeats.

Primers for q-RT-PCR were found in the literature and some were designed using primer blast (https://www.ncbi.nlm.nih.gov/tools/primer-blast/), (Ye et al., 2012). Primers were designed to generate 100 bp products with a melting temperature of between 59 and 62 ^o^C. Primers used are listed in Table 4.

**Table 4:** Q-RT PCR Primers used for *X. tropicalis* embryos. miRCURY LNA miRNA PCR primers, Qiagen. mRNA primers were ordered as standard oligos.

Primer name	Sequence 5’ to 3’	Product code/Accession number for design
xtr-miR-196a	UAGGUAGUUUCAUGUUGUUGG	YP02103491 (Qiagen)
ipu-miR-219a	AGAAUUGUGCCUGGACAUCUGU	YP02101832 (Qiagen)
U6 snRNA	CTCGCTTCGGCAGCACA	YP00203907 (Qiagen)
hsa-miR-219a-5p	UGAUUGUCCAAACGCAAUUCU	YP00204780 (Qiagen)
EEF1Alpha F X.tr	CCCAACTGATAAGCCTCTGC	PMID 23559567(Dhorne-Pollet et al., 2013)
EEF1Alpha R X.tr	CATGCCTGGCTTAAGGACAC	PMID 23559567 (Dhorne-Pollet et al., 2013)
Sox10 ​F X.tr	GATGGGTCCTCTGAAGCTGA	Self designed NM_001100221.1
Sox10 ​R X.tr	GGTAGGGGGTCCATGACTTT	Self designed NM_001100221.1
Snail2 F X.tr	CCCCATTCCTGTATGAGCGG	PMID: ​32713114, (Wang et al., 2020)
Snail2 R X.tr	TGAAGCAGTCCTGTCCACAC	PMID: ​32713114, (Wang et al., 2020)
Xhe2 F2 X.tr	CGCCACCTCTTTTCCCATTCA	Self designed NM_001044399.1
Xhe2 R2 X.tr	TTTGGGCCACAGACACTCCTT	Self designed NM_001044399.1
Pax3 F X.tr	TACAGCATGGAGCCTGTCAC	PMID: ​24055059 (Gentsch et al., 2013)
Pax3 R X.tr	TCCTTTATGCAATATCTGGCTTC	PMID: ​24055059 (Gentsch et al., 2013)
EEF1Alpha F X.la	ACCCTCCTCTTGGTCGTTTT	PMID: (24360908) (Bae et al., 2014)
EEF1Alpha R X.la	TTTGGTTTTCGCTGCTTTCT	PMID: (24360908) (Bae et al., 2014)
Eya1 F X.la	ATGACACCAAATGGCACAGA	PMID: 17409353 (Hong and Saint-Jeannet, 2007)
Eya1 R X.la	GGGAAAACTGGTGTGCTTGT	PMID: 17409353 (Hong and Saint-Jeannet, 2007)
Pax3 F X.la	CAAGCTCACAGAGGCGCGAGT	PMID: 29038306 (Figueiredo et al., 2017)
Pax3 R X.la	AGCTGGCATAGCTGCAGGAGG	PMID: 29038306 (Figueiredo et al., 2017)
Sox10 ​F X.la	CTATTACTGACACACGACGGAGC	PMID (32494672) (Scerbo and Monsoro-Burq, 2020)
Sox10 ​R X.la	ACCTCTCATCCTCTGAATCCTGC	PMID(32494672) (Scerbo and Monsoro-Burq, 2020)
Snail2 F X.la	CACACGTTACCCTGCGTATG	PMID: 29038306 (Figueiredo et al., 2017)
Snail2 R X.la	TCTGTCTGCGAATGCTCTGT	PMID: 29038306 (Figueiredo et al., 2017)
Xhe2 F X.la	CATGTCTAATGGCGGTTGTG	PMID: (24360908) (Bae et al., 2014)
Xhe2 R X.la	TGCTGGATGATCCCCATATT	PMID: (24360908) (Bae et al., 2014)

### 4.8 Whole-mount *in situ* hybridisation & Riboprobe synthesis

Standard *in situ* hybridisations and probe synthesis were carried out according to (Harrison et al., 2004; Monsoro-Burq, 2007; Sive et al., 2007). In brief, Whole-mount *in situ* hybridisation (WISH) with LNA probes was carried out with probes hybridised at 50^o^C at a concentration of 1 μg/mL, overnight with embryos, before stringency washes with graded SSC washes, blocking and incubation overnight with anti-DIG antibody fragments (Roche, 11093274910). MAB washes then removed unbound antibody prior to colour development with NBT/BCIP. The LNA WISH experiments were carried out according to Ahmed et al. (2015) and Sweetman et al. (2006). Probe synthesis experiments involved digestion of plasmid with appropriate restriction enzyme to produce antisense product which was then transcribed by respective polymerase, (Table 5).

**Table 5:** Riboprobe synthesis and capped RNA plasmids information.

Clone name	Antibiotic resistance	Backbone	Antisense RE	Antisense Polymerase	Source
Pax3	Ampicillin	pBSK	BglII	SP6	M.G. Sargent
Sox10	Ampicillin	pBSK	EcoRI	T3	J.P. Saint-Jeannet
Snail2	Ampicillin	pCS107	EcoRI/BamHI	T7	EXRC
Xhe2	Ampicillin	pBSK	XbaI	T7	A.H.Monsoro-Burq
GFP2	Ampicillin	pCS	NotI (sense)	SP6 (sense)	M. Walmesly
LacZ	Ampicillin	pCS	NotI (sense)	SP6 (sense)	M. Walmsely
