# Supplementary material for: An efficient miRNA knockout approach using CRISPR-Cas9 in Xenopus
Source: Dev Biol. 2022 Mar;483:66–75. doi: 10.1016/j.ydbio.2021.12.015 (PMC8865746; doi:10.1016/j.ydbio.2021.12.015)
Supplement: Multimedia component 1 [file mmc1.docx]

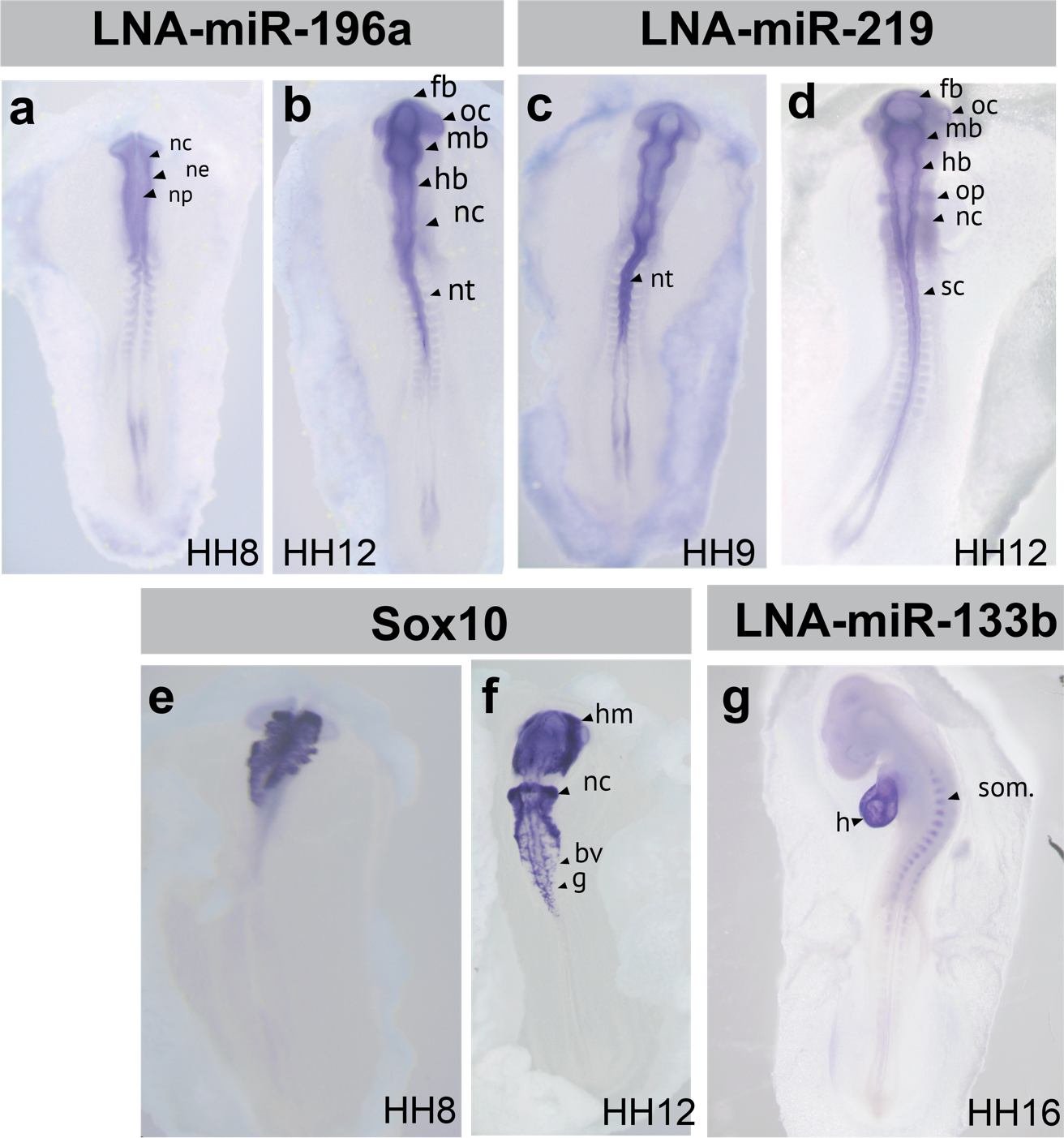


**Supplementary Figure- 1: Expression profile of: Sox10, miR-196a, miR-219 and miR-133b in chick embryos.** Embryos staged according to Hamilton & Hamburger. LNA-miR-196a (a-b), and LNA-miR-219 (c-d), both show expression in NC tissues, and neural tissues including brain structures. Sox10 expression can clearly be seen in NC at HH12 (e-f). Sox10 is expressed in the blood vessels, ganglia, head mesenchyme but also clearly in the NC, as indicated by black arrows. MiRNA *in situ* hybridisation experiments were carried out with LNA probes. LNA-miR-133b clearly shows miR-133b expression in the heart and somites at HH16 (g). Abbreviations: bv- blood vessels, fb- forebrain, h- heart, g- ganglia, hb- hindbrain, hm- head mesenchyme, mb- midbrain, nc- NC, ne- neural, np- neural plate, nt- neural tube, oc- optic cup, op- otic placode, sc- spinal cord, som- somites.


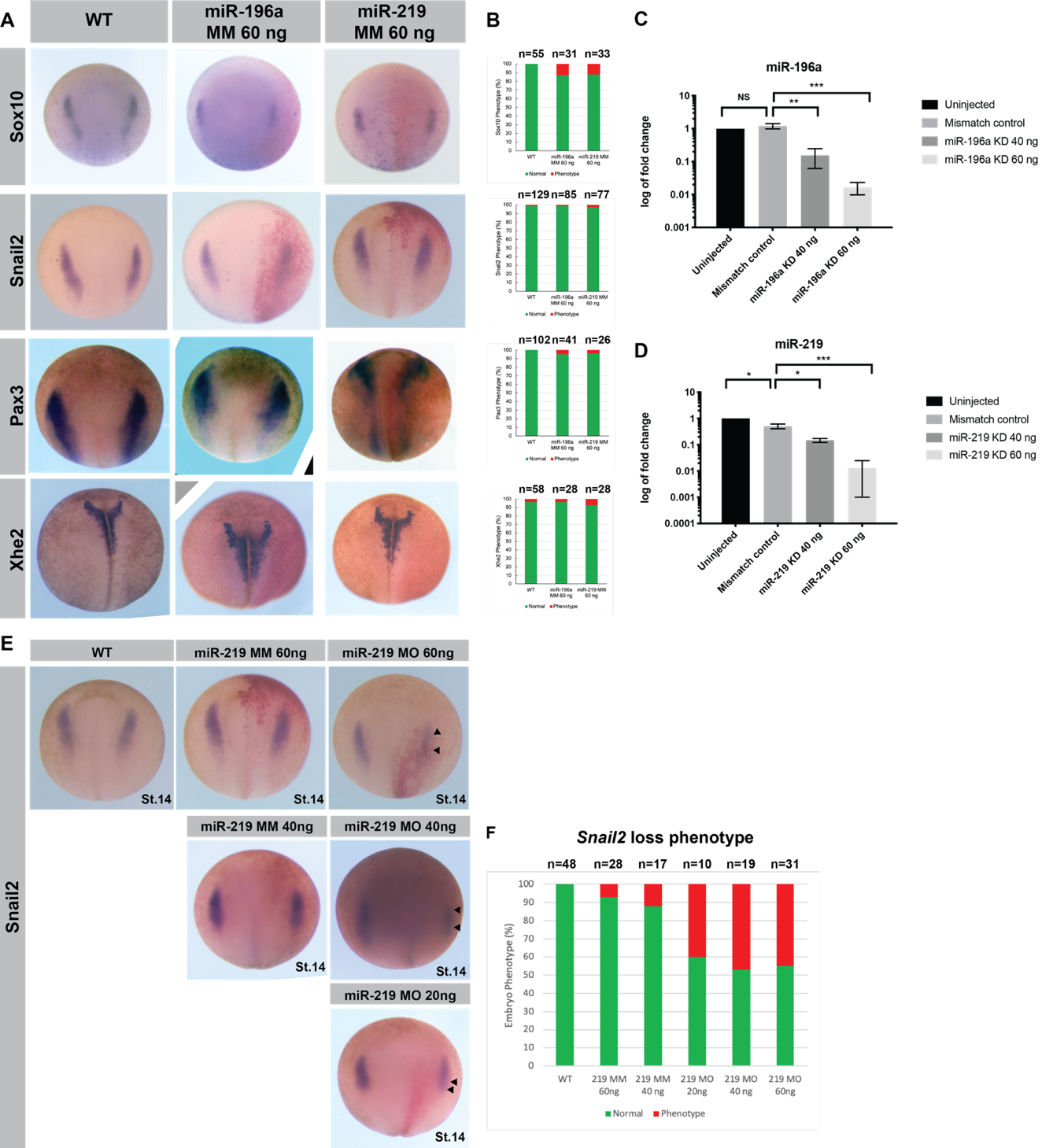


**Supplementary Figure- 2: Control and validation experiments for morpholino-mediated miRNA KD.** (A) Control experiments with morpholinos, showing WT, control morpholino “MM”- mismatch, for miR-196a and miR-219. X. *laevis* embryos were injected at 4-cell stage into the right dorsal blastomere with LacZ cRNA tracer. (B) Shows phenotype count data, assessed as in Fig.3. (C-D) Q-RT-PCR validation of morpholino dose-response to show morpholino specificity. One-way Anova with post-hoc Tukey tests show statistical significance, with p=<0.05 for “*” as the threshold, p=<0.01 “**” and p=<0.001 for “***”, and ns= not significant.


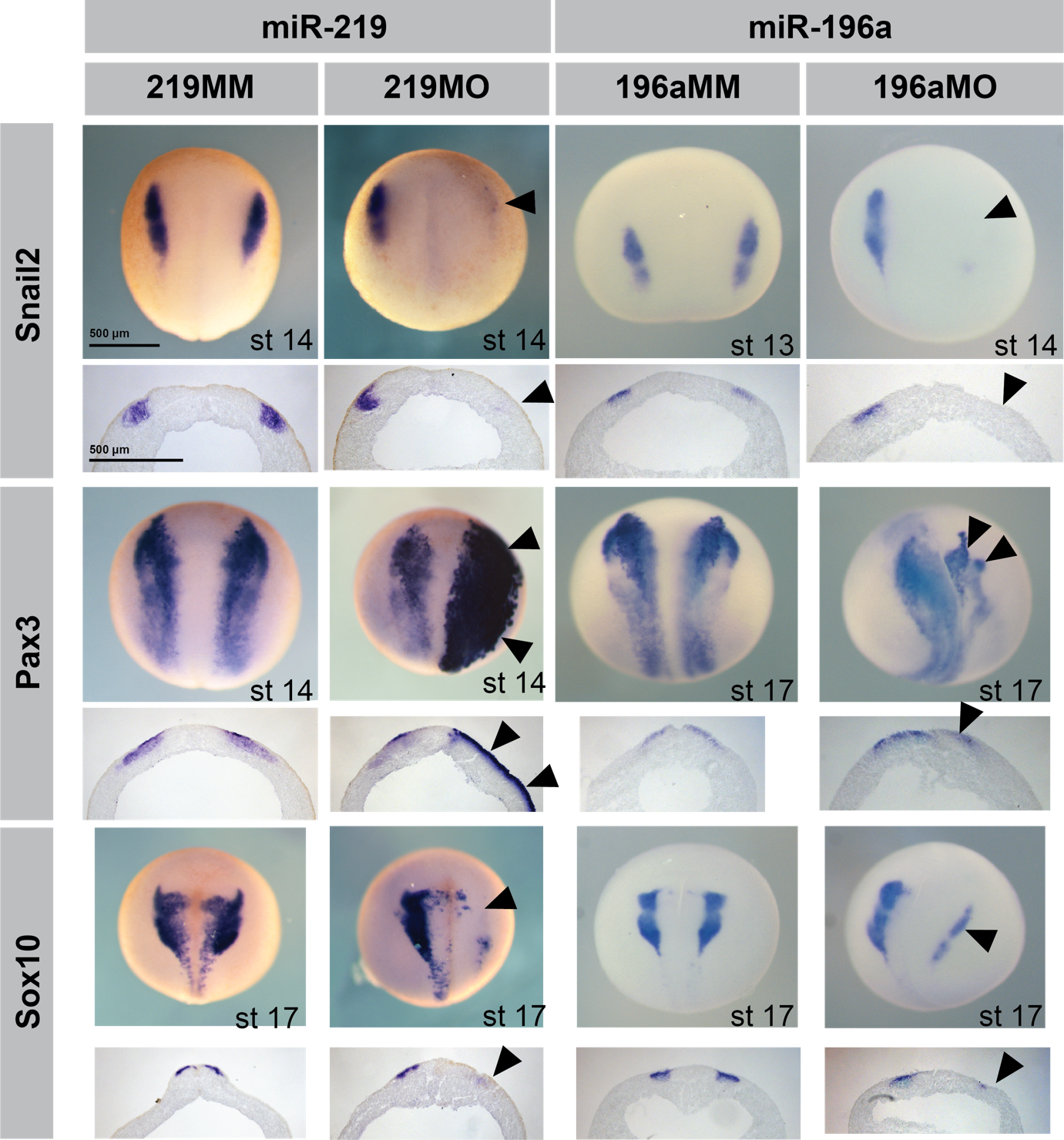


**Supplementary Figure- 3: Morpholino-mediated KD of miRNAs and expression of key NC and NP markers.** 219MM= miR-219 mismatch-morpholino 60 ng, 219MO= miR-219 morpholino 60 ng, 196MM= miR-196a mismatch-morpholino 60 ng, 196MO= miR-196a morpholino 60 ng. GFP cRNA tracer was used, and positive embryos were selected for sectioning. NC markers Snail2 and Sox10 showed clear reduction in expression following MO-mediated miRNA KD. In comparison to this, there were contrasting results observed for neural plate marker Pax3 following MO-mediated miRNA KD. MiR-219 KD showed marked expansion in superficial ectoderm region, whereas miR-196a KD showed altered expression. This suggests NC and NP are affected differently by miRNA KD.

**
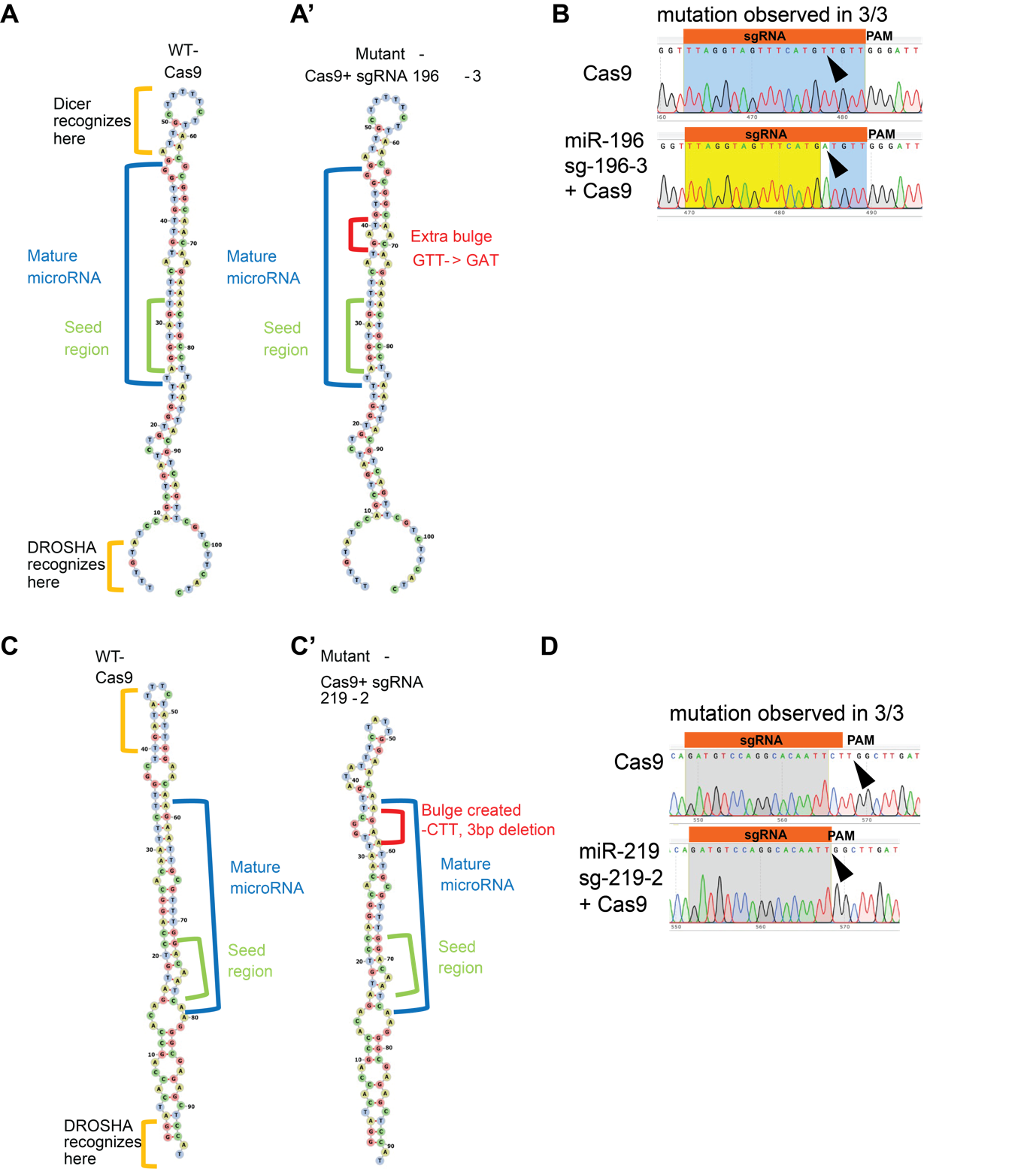
**

**Supplementary Figure- 4: Modelling individual sgRNA miRNA mutations.** Individual sgRNAs were used to disrupt the mature miRNA for miR-196a and miR-219. For miR-196a (A-A’) and miR-219 (C-C’) the wild-type and mutant stem-loops highlight the mature miRNA (blue bars), seed region (green bars), and DROSHA AND DICER recognition sites (yellow bars). The mutant regions in (A’) and (C’) are highlighted by red bars. These modelled mutations can be seen in the sequence data chromatograms in (B) for miR-196a mutant and (D) for miR-219 mutant.


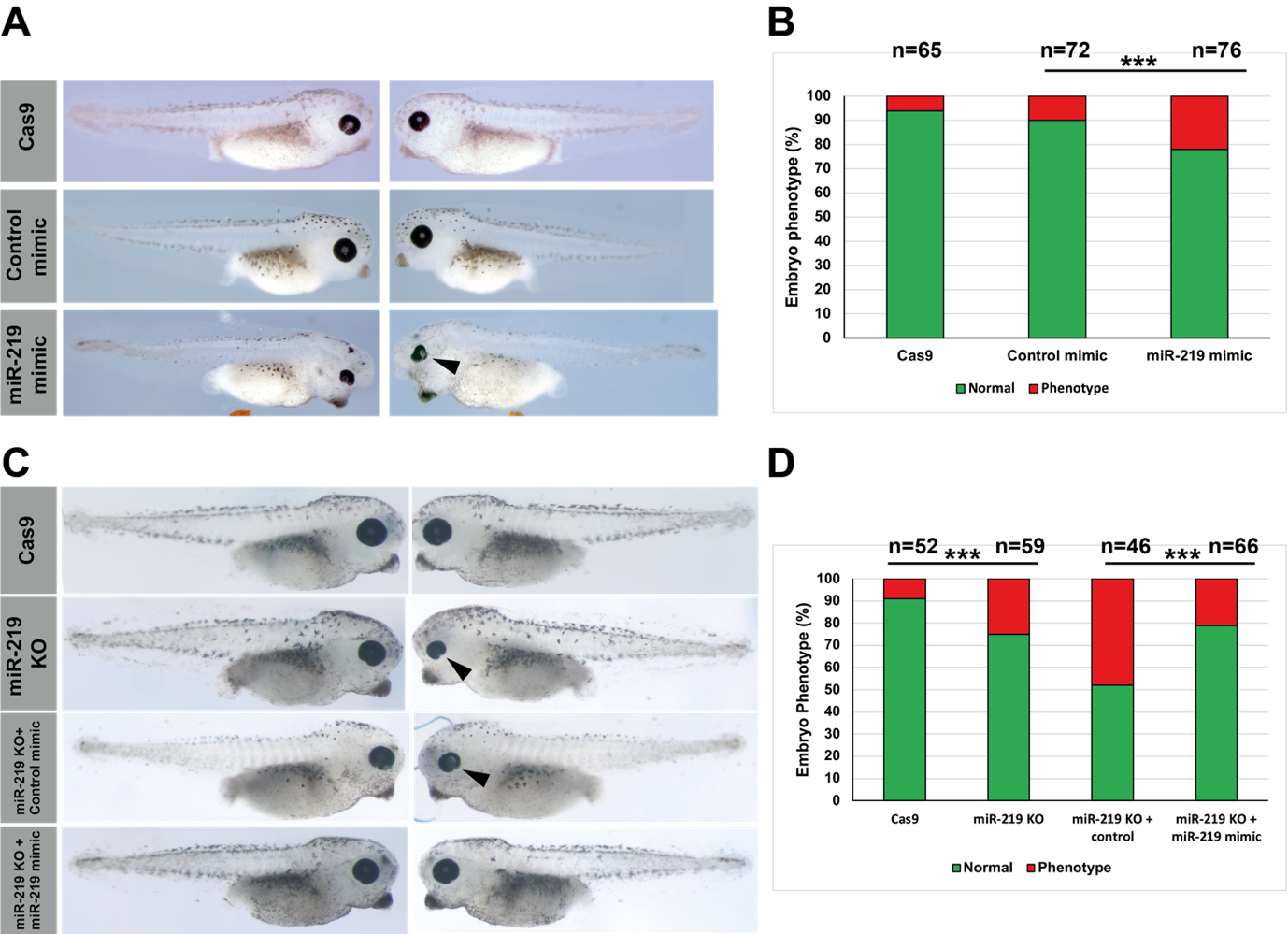


**Supplementary Figure- 5: Novel miRNA KO rescue experiment.** To rescue loss of miRNA following KO with CRISPR-Cas9 miRNA mimic (miR-219) and control miRNA mimic (Cel-miR-39-3p) were overexpressed in embryos and the phenotypes observed (A). Craniofacial phenotypes were observed following overexpression of miR-219 mimic, as indicated by black arrow, this phenotype was significantly more prevalent than in control groups (B); P value for control mimic vs miR-219 mimic = 0.00041. For the miR-219 KO rescue, Cas9 injected group was used as a negative control for the miR-219 KO, and miR-219 KO + control mimic was used as a control for miR-219 KO + miR-219 mimic (rescue). MiR-219 KO and miR-219 KO + control mimic show the expected craniofacial phenotypes following loss of miR-219. This craniofacial phenotype is denoted by black arrows and is rescued with use of miR-219 mimic in conjunction with CRISPR reagents (C). The rescue seen following use of miR-219 mimic compared to control mimic was statistically significant. P values for Cas9 vs miR-219 KO= 0.0008. P value for miR-219 KO + control mimic vs miR-219 KO + miR-219 mimic= 0.001. Developing X. tropicalis embryos were injected at the 4-cell stage into 1 blastomere with 4.2 nL volume containing: Cas9 protein 2.4 mM + 5ng GFP cRNA, Control miRNA mimic 11 µM + 5 ng GFP cRNA, miR-219 mimic + 5 ng GFP cRNA, miR-219 sgRNAs 300 pg + Cas9 protein 2.4 mM + 5 ng GFP cRNA, miR-219 sgRNAs 300 pg + Cas9 protein 2.4 mM + 5 ng GFP cRNA + control miRNA mimic 11 µM (miR-219 KO + control mimic), miR-219 sgRNAs 300 pg + Cas9 protein 2.4 mM + 5 ng GFP cRNA + miR-219 mimic 11 µM (miR-219 KO + miR-219 mimic). Injected embryos were screened for GFP positive expression to be included in the study. Statistical significance was calculated using chi-squared.

~~
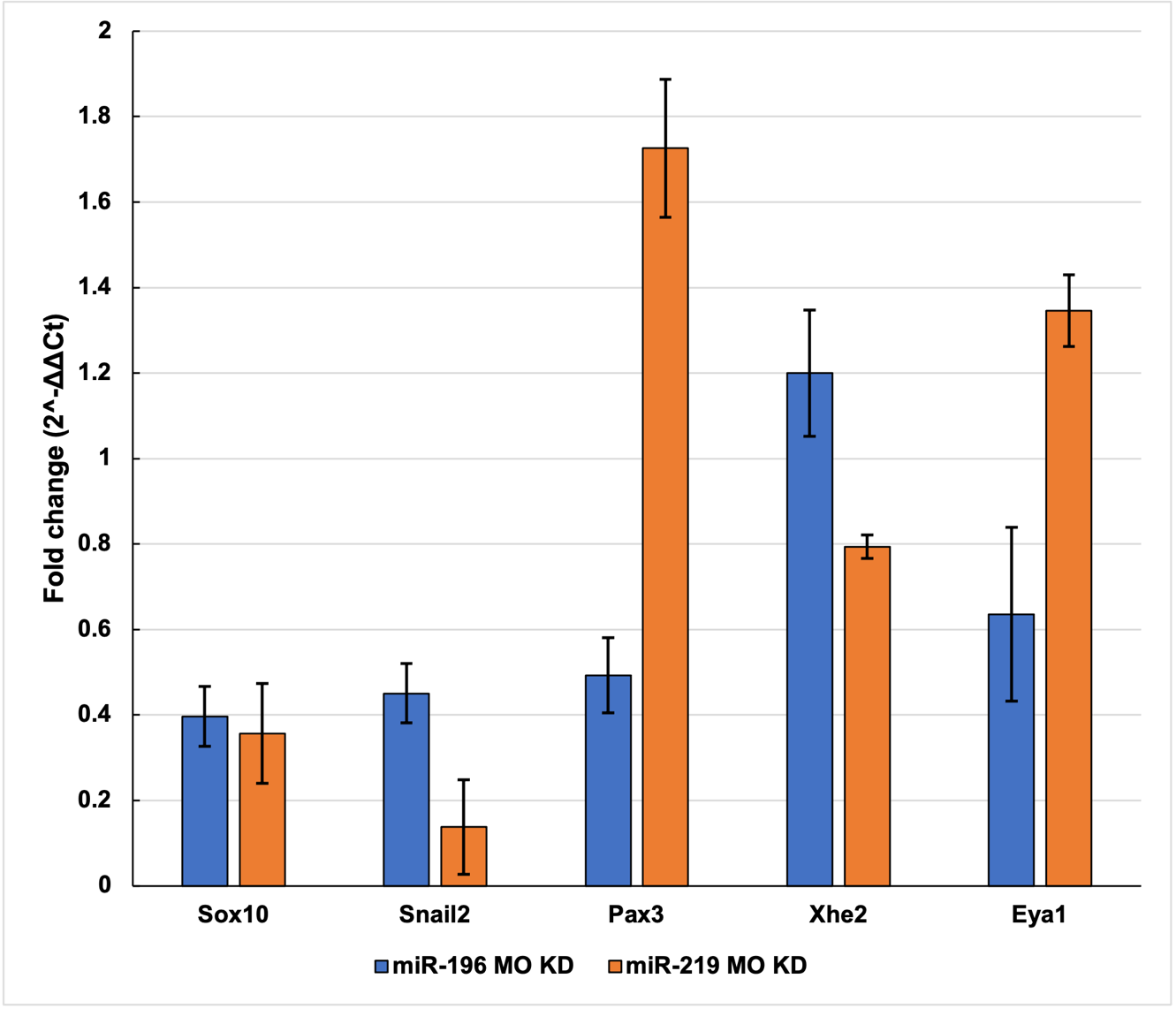
~~

**Supplementary Figure** **6**- Q-RT-PCR validation of mRNA gene expression following loss of miR-196a and miR-219. NC markers Sox10 and Snail2 show reduction in expression following loss of miRNAs. Pax3 showed loss for miR-196a KD and enrichment following miR-219 KD. HG marker Xhe2 showed small enrichment following miR-196a KD and a small reduction in expression following miR-219 KD. Eya1 showed strong loss in expression following miR-196a KD and enrichment following loss of miR-219. Experiments were performed with biological and technical triplicate. Bars depict the mean of expression and error bars show mean +/- S.E.M.
